# Effect of Amphotericin B on the Thermodynamic Properties and Surface Morphology of the Pulmonary Surfactant Model Monolayer during Respiration

**DOI:** 10.3390/molecules28124840

**Published:** 2023-06-18

**Authors:** Juan Wang, Jia Wang, Xinzhong Wang, Zhen Wang

**Affiliations:** 1 Shaanxi Engineering Research Center of Controllable Neutron Source, School of Electronic Information, Xijing University, Xi’an 710123, China; 2 Xi’an Key Laboratory of Human-Machine Integration and Control Technology for Intelligent Rehabilitation, School of Computer Science, Xijing University, Xi’an 710123, China

**Keywords:** pulmonary surfactant, amphotericin B, Langmuir monolayer, atomic force microscopy

## Abstract

During the COVID-19 pandemic, the treatment of pulmonary fungal infection faced noteworthy challenges. Amphotericin B has shown promising therapeutic effects as an inhalation treatment for pulmonary fungal infections, especially those associated with the COVID-19 virus, due to its rare resistance. However, because the drug frequently produces renal toxicity, its effective dose is limited in clinical use. In this work, the DPPC/DPPG mixed monolayer was used as the pulmonary surfactant monolayer to study the interaction between amphotericin B and the pulmonary surfactant monolayer during inhalation therapy using the Langmuir technique and atomic force microscopy. The effects of different molar ratios of AmB on the thermodynamic properties and surface morphology of the pulmonary surfactant monolayer at different surface pressures was evaluated. The results showed that when the molar ratio of AmB to lipids in the pulmonary surfactant was less than 1:1, the main intermolecular force was attractive at a surface pressure greater than 10 mN/m. This drug had little effect on the phase transition point of the DPPC/DPPG monolayer, but decreased the height of the monolayer at 15 mN/m and 25 mN/m. When the molar ratio of AmB to lipids was greater than 1:1, the intermolecular force was mainly repulsive at a surface pressure greater than 15 mN/m, and AmB increased the height of the DPPC/DPPG monolayer at both 15 mN/m and 25 mN/m. These results are helpful in understanding the interaction between the pulmonary surfactant model monolayer and different doses of drugs at various surface tensions during respiration.

## 1. Introduction

Invasive fungal infections, especially pulmonary fungal infections, have become a growing public health problem due to the use of immunosuppressants in tumor therapy, the mixed use of antibiotics, and the increase in the population with low immunity [1]. Although they are less common than bacterial or viral infections, pulmonary fungal infections have a serious impact on the morbidity and mortality of patients [2]. In recent years, during the global COVID-19 pandemic, bacterial and fungal co-infection has become one of the multiple factors affecting the morbidity and mortality of patients infected with COVID-19, especially in patients with acute respiratory distress syndrome [3,4]. Tissue damage caused by SARS CoV-2 may promote the invasion of symbiotic yeast, leading to deep invasive fungal infection [5]. Pulmonary aspergillus infection is one of the major factors leading to shortened survival of patients severely infected with COVID-19. Data show that the incidence of pulmonary aspergillus infection in critically ill patients infected with COVID-19 is 3.3–34.4%, and the all-cause mortality of patients infected with pulmonary aspergillus is significantly higher than that of patients without aspergillus infection [6]. The incidence of Candida in critical cases of COVID-19 increases, and the mortality rate remains as high as 83%, even after the antifungal therapy [6]. Meanwhile, mechanical ventilation in patients with acute respiratory distress syndrome increases the incidence of pulmonary fungal infection. Some reports have shown that mechanical ventilation for longer than 48 h can causes a Candida infection in 50% of those diagnosed with ventilator-associated pneumonia [7,8]. Therefore, in the context of the COVID-19 epidemic, pulmonary fungal infection during the treatment of this disease is a noteworthy challenge.

**Figure 1:**
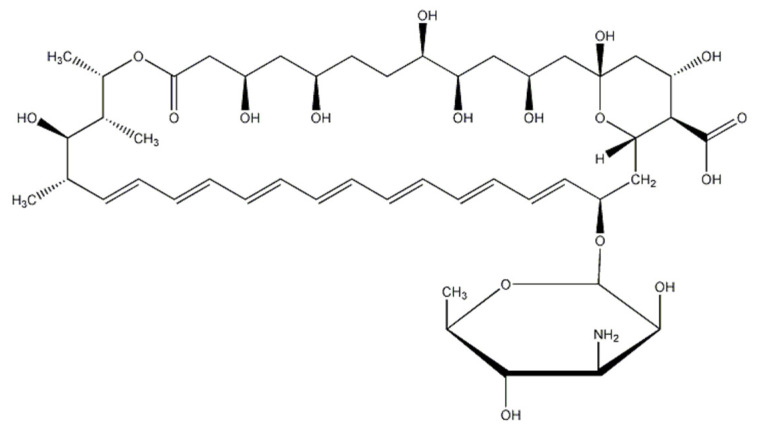
The chemical formula of an AmB molecule.

However, in the treatment of fungal infections, most drugs are prone to resistance, and have limited antifungal effects [9]. Amphotericin B is the most-used drug of choice in clinical use, and it is the gold standard for treating systemic fungal infections due to its rare drug resistance [10]. It consists of a heptaene macrolide skeleton, with a functional carboxyl group at position C16 and a mycosamine sugar appendage at position C19 (Figure 1). AmB has been administered as inhalation therapy for pulmonary fungal infections [11], especially for the lung diseases associated with COVID-19 infection, producing good results [12]. However, due to its susceptibility to causing both cell membrane and renal toxicity, it is subject to dose limitation in clinical practice, which impacts its therapeutic effect. How to effectively improve the efficacy of AmB in the treatment of pulmonary fungal infections and reduce the toxicity of the drug is a problem of concern. The fundamental solution to this problem is to identify the interaction between the AmB drug and the pulmonary surfactants in the lungs after inhalation.

The alveoli expand during inspiration, resulting in a low surfactant concentration and increased surface tension. At the end of inspiration (1.75 s), the surface tension value peaks at 25 mN/m. The alveoli contract during exhalation, causing the surfactant concentration to increase and the surface tension to decrease to a value near zero. At the end of exhalation (4.75 s), the surface tension gradually drops to about 0 mN/m [13,14]. It should be emphasized that the pulmonary surfactant can maintain a low alveolar surface tension and prevent lung collapse [15], and its dysfunction has been associated with many airway diseases, such as asthma [16], cystic fibrosis [17], and COVID-19 [18].

When AmB is inhaled into the lungs, the mechanism of the drug affecting the structure and properties of the lung surfactant membranes during respiration is still unclear, requiring further in-depth study. However, there is currently no technical method for studying the interface behavior of the pulmonary surfactant in vivo. The Langmuir technique is a popular in vitro method used to study the interactions of drug with pulmonary surfactants under simulated physiological conditions, helping us to understand the interface behavior of pulmonary surfactants [19]. Pulmonary surfactants cover the surface of epithelial cells throughout the respiratory region [20] and are composed of surfactant lipids and surfactant proteins. In the lung surfactants of human, lipids are the main components, accounting for about 90% by weight. In all lipids, phosphatidylcholine (PC) lipids make up about 80%, and the saturated dipalmitoyl phosphatidylcholine (DPPC), which reduces surface tension, comprises about 41% [21,22,23]. The dipalmitoyl phosphatidylglycerol lipid (DPPG) makes up about 10%, and its function is to enhance the fluidity of the monolayer film [24]. The mixed monolayer composed of DPPC and DPPG (4:1, mol:mol), which has been widely used to study the interaction between the drug, the drug-carrying nanomaterials, and the pulmonary surfactant model monolayer, is the typical model of the real pulmonary surfactant model monolayer [25,26].

In this paper, the Langmuir technique was used to study the effects of different molar ratios of AmB on the phase behavior, thermodynamic parameters, elastic modulus, and relaxation of the DPPC/DPPG monolayers at the air–water interface. The Langmuir–Blodgett deposition technique was used to transfer mixed monolayers onto the mica at different surface pressures to study the effect of AmB on the microstructure of the monolayers using atomic force microscopy. The results are helpful for understanding the molecular mechanism of the interaction of AmB with the pulmonary surfactant monolayer during respiration.

## 2. Results and Discussions

### 2.1. Parameter Analysis of Surface Pressure—Mean Molecular Area (π−A) Isotherm

**Figure 2:**
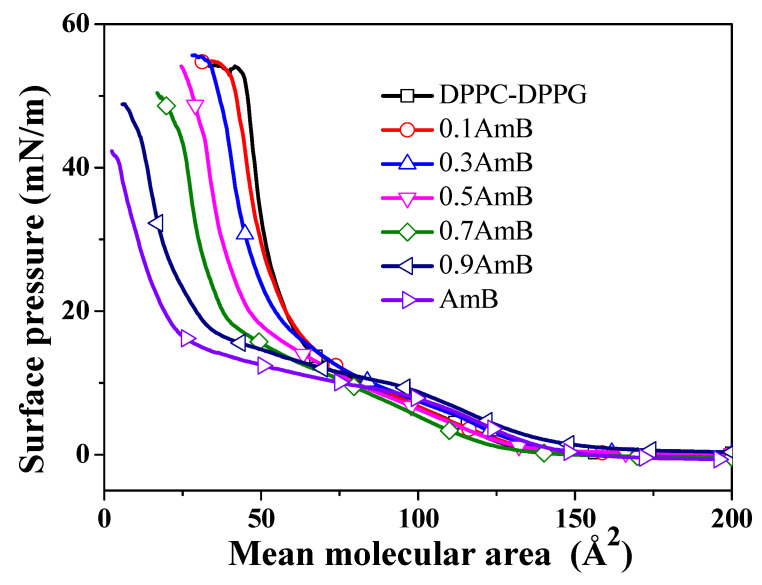
The surface pressure–mean molecular area isotherms of the AmB/DPPC/DPPG mixed monolayers on the air–water interface at 35.0 ± 0.5 °C.

The π−A isotherms, which gradually rise with the increase in surface pressure, are shown in Figure 2. At high surface pressures, the mean molecular area decreases with the increase in the molar ratio of AmB. The trend of the isotherm for the DPPC/DPPG monolayer is similar to that in observed in the literature [27] and in our previous works [28,29]. At the air–water interface, the monolayer may exist in different states, such as gaseous, liquid-expanded (LE), liquid-condensed (LC), solid, and intermediate or transition films, at different surface pressures, a behavior which, in many respects, is analogous to those observed in three-dimensional systems [30,31]. At lower surface pressures, an obvious inflection point of the π−A isotherm is relative to the change of phase state. At higher surface pressures, an obvious inflection point indicates that the monolayer gradually begins to collapse. There are three characteristic parameters on the π−A isotherm, i.e., the liftoff area $A_{L}$, the limiting area $A_{\infty }$, and the surface pressure $\pi_{C}$ at collapse [30,31]. The $A_{L}$ is the molecular occupation area where the rising isotherm barely emerges relative to the baseline. The $A_{\infty }$ is an empirical parameter approximating the mean molecular cross-sectional area, which is calculated according to the π−A isotherms by extrapolating the slope of the isotherm in its steepest range in relation to the zero-surface pressure [32]. The mean molecular area at collapse is marked as $A_{C}$.

From the characteristic parameters on the π−A isotherm shown in Table 1, the $A_{L}$ values of the DPPC/DPPG monolayers increased after adding different molar ratios of AmB. However, with the increase in the molar ratio of AmB, the change in the $A_{L}$ value does not show an obvious pattern. Driven by the lateral pressure of the barrier, although the average molecular area is reduced, the distance between molecules is still significant, and they are in the gaseous state. In this case, the intermolecular force does not dominate, which is also indicated by the excess Gibbs free energy values near 1 mN/m. The parameters $A_{\infty }$, $\pi_{C}$, and $A_{C}$ show a consistent pattern and decrease gradually with the increase in the molar ratio of AmB. However, the $A_{C}$ values when $x_{AmB}=1\;or$ 0.9 are very small. This may be due to the fact that the AmB molecules form complexes in the process of monolayer compression, and with the increase in surface pressure, the AmB complexes are gradually extruded, escaping to the subphase.

The molecular packing of AmB molecules in the DPPC/DPPG monolayer can be analyzed in terms of the additivity rule:

(1) $${A_{\infty }^{*}=x_{1}A}_{\infty -DPPC/DPPG}+x_{2}A_{\infty -AmB}$$

where $A_{\infty }^{*}$ is the theoretical limiting area when the lipids and AmB molecules are ideally mixed, and $x_{1}$, $x_{2}$ are the molar fractions of the DPPC/DPPG mixture and the AmB molecules. When the molar ratio of AmB gradually increases from 0.1 to 0.5, the theoretical limiting areas of the mixed system are 58.98 ${Å}^{2}$, 53.21 ${Å}^{2}$, and 47.43 ${Å}^{2}$, respectively, which are then reduced by about 2.65~3.55 ${Å}^{2}$ compared with the experimental values. However, when the molar ratio of AmB is 0.9, the theoretical value is 0.68 ${Å}^{2}$ lower than the experimental value. This may be related to the aggregation of AmB molecules to form complexes. The exception is that when the molar ratio of AmB is 0.7, the theoretical value is greater than the experimental value, but the difference (0.86 ${Å}^{2}$) is close to that occurring when $x_{AmB}$ = 0.9. This may be because a small number of lipid molecules or AmB complexes are pushed out of the interface during the compression of the monolayer.

### 2.2. Thermodynamic Analysis

The interaction between the three components in a mixed monolayer can be evaluated quantitatively by the excess Gibbs energy ($G^{E}$) at a constant surface pressure and temperature. The $G^{E}$ value is positive or negative, which corresponds to a repulsive force or an attractive force between the molecules. The $G^{E}$ value can be calculated by the formula [33]:

(2) $$G^{E}=\int_{0}^{\pi }\left[A_{123}-\left(x_{1}A_{1}+x_{2}A_{2}+x_{3}A_{3}\right)\right]d\pi$$

where $x_{1}$, $x_{2}$, $and\;x_{3}$ are the molar fractions of DPPC, DPPG and AmB in the mixed monolayer, $A_{1}$, $A_{2}$, and $A_{3}$ are the mean molecular area of the pure DPPC, DPPG and AmB monolayer, respectively, and $A_{123}$ is the mean molecular area of the AmB/DPPC/DPPG mixed monolayer.

Another thermodynamic parameter, the Gibbs energy of mixing (${∆}_{mix}G$), can be used to quantitatively evaluate the stability of the monolayers. A positive ${∆}_{mix}G$ value indicates the instability of the monolayer, and a negative value indicates stability. The ${∆}_{mix}G$ value can be calculated by the formula [34]:

(3) $${∆}_{mix}G=G^{E}+RT\left(x_{1}\ln x_{1}+x_{2}\ln x_{2}+x_{3}\ln x_{3}\right)$$

where *R* and *T* are the universal gas constant and temperature, respectively.

**Figure 3:**
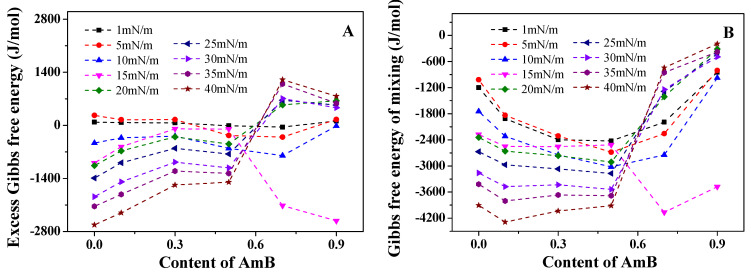
The excess Gibbs free energy ($G^{E}$, (**A**)) and Gibbs free energy of the mixing (${∆}_{mix}G$, (**B**)) of the DPPC/DPPG mixed monolayers with the different molar radios of AmB at different surface pressures.

At low surface pressures (1 and 5 mN/m), the $G^{E}$ values of the DPPC/DPPG mixed monolayer are positive, suggesting that the intermolecular force is repulsive. At other surface pressures (10, 15, 20, 25, 30, 35, and 40 mN/m), the $G^{E}$ values are negative, indicating that the intermolecular force is attractive (Figure 3A). The absolute value of $G^{E}$ increases with the increase in the surface pressure, indicating that the strength of the intermolecular force is enhanced at higher surface pressures. When the molar ratio of AmB ($x_{AmB}$) is 0.1, the variation of the $G^{E}$ value with the surface pressure is similar to that of the DPPC/DPPG mixed monolayer. When $x_{AmB}$ is 0.3, the absolute value of negative $G^{E}$ suddenly decreases at 15 mN/m, which is different from that at $x_{AmB}=0.1$. When $x_{AmB}$ is 0.5, the $G^{E}$ values are all negative at the surface pressure mentioned in this work. With the increase in the surface pressure, the absolute value of $G^{E}$ first increases at 1~15 mN/m, then decreases significantly at 15 mN/m, and then increases gradually at 15~40 mN/m. When $x_{AmB}$ is 0.7, the $G^{E}$ value is negative at a surface pressure lower than 15 mN/m, and it suddenly becomes positive at a surface pressure higher than 15 mN/m. Whether the surface pressure is lower or higher than 15 mN/m, the absolute value of $G^{E}$ increases with the increase in the surface pressure. This indicates that the intermolecular force of the AmB/DPPC/DPPG mixed monolayer is attractive at a surface pressure lower than 15 mN/m, and it is repulsive at a surface pressure higher than 15 mN/m. When $x_{AmB}$ is 0.9, the $G^{E}$ value is only negative at 10 mN/m and 15 mN/m. The absolute value of $G^{E}$ is the greatest at 15 mN/m, meaning that the intermolecular attraction reaches the maximum. However, when the surface pressure exceeds 15 mN/m, the intermolecular force suddenly changes into a repulsive force, which may be due to the interaction between the polyol chain of the AmB and lipid chains.

The ${∆}_{mix}G$ value is negative for all components and all surface pressures involved (Figure 3B), indicating that the mixed system is stable. When $x_{AmB}$ is 0.1, 0.3, and 0.5, the absolute value of ${∆}_{mix}G$ of the DPPC/DPPG monolayers increases due to the addition of AmB, indicating that the AmB/DPPC/DPPG mixed monolayer is more stable than the DPPC/DPPG monolayer, caused by the interaction between AmB and the lipid molecules. However, when $x_{AmB}$ is 0.7 and the surface pressure is 20~40 mN/m, the stability of the DPPC/DPPG mixed monolayer is reduced due to the addition of AmB, which is similar to that when $x_{AmB}$ is 0.9, at all pressures except 15 mN/m. This indicates that the effect of AmB on the stability of the DPPC/DPPG monolayer is directly related to the molar ratio of AmB in the system. When the molar ratio of AmB is larger, the stability of the mixed monolayer at 20~40 mN/m will decline. Interestingly, when $x_{AmB}$ is 0.7 and 0.9, the absolute value of ${∆}_{mix}G$ increases significantly at a surface pressure of 15 mN/m, which indicates that the AmB/DPPC/DPPG mixed monolayer is the most stable.

### 2.3. The Modulus of Elasticity

The modulus of elasticity is an important parameter characterizing the compressibility of the monolayers, and a large modulus of elasticity corresponds to a low compressibility of the monolayer. This can be calculated using the data from a surface pressure–mean molecular area isotherm, as follows [35,36,37]:

(4) $$C_{s}^{-1}=-A{\left(\partial \pi /\partial A\right)}_{T}$$

where *s* is the cross-sectional area of the monolayer, *A* is the mean molecular area, and *π* is the surface pressure of the monolayer.

**Figure 4:**
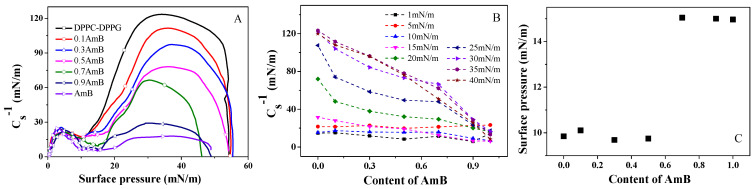
The modulus of elasticity ($C_{s}^{-1}$)–surface pressure curves of the DPPC/DPPG mixed monolayers at the different molar ratios of AmB (**A**), the plot of the $C_{s}^{-1}$ value versus the molar ratio of AmB at different surface pressures, (**B**) and the surface pressure corresponding to the minimum of $C_{s}^{-1}$ (**C**).

From Figure 4A, it can be noted that the AmB drug reduces the maximum $C_{s}^{-1}$ value for the DPPC/DPPG monolayer, and the maximum $C_{s}^{-1}$ value is smallest when $x_{AmB}$ is 0.9, which indicates that AmB increases the compressibility of the DPPC/DPPG monolayer.

According to Figure 4B, when the surface pressure is lower than 15 mN/m, there is no significant pattern in the change of the $C_{s}^{-1}$ value as the AmB molar ratio increases. When the surface pressure is greater than 15 mN/m, the $C_{s}^{-1}$ value gradually decreases with the increase in the AmB molar ratio. When $x_{AmB}$ is 0.1, 0.3, and 0.5, the $C_{s}^{-1}$ value reaches the maximum at 35 mN/m. When $x_{AmB}$ is 0.7 and 0.9, the $C_{s}^{-1}$ value reaches the maximum at 30 mN/m. In the range of 15~35 mN/m for $x_{AmB}$ = 0.1, 0.3, and 0.5, and in the range of 15~30 mN/m for $x_{AmB}$ = 0.7 and 0.9, the $C_{s}^{-1}$ value increases with the increase in surface pressure.

The minimum $C_{s}^{-1}$ value indicates a significant phase transition in the monolayer [38]. The surface pressures corresponding to the point at which the phase transition occurs is shown in Figure 4C. The $C_{s}^{-1}$ value of the DPPC/DPPG monolayer appears to be minimal at near 10 mN/m, which does not changed dramatically when the molar ratio of AmB added in the mixed system is 0.1, 0.3, and 0.5. However, when the molar ratios of AmB are 0.7 and 0.9, the minimum $C_{s}^{-1}$ for the mixed monolayer appears at near 15 mN/m. This may be due to the phase transition of the pure AmB monolayer at 15 mN/m, which corresponds to the obvious plateau on the π−A isotherm (Figure 2). This may be related to the orientation of the AmB molecules on the lipid monolayers.

### 2.4. Relaxation of the DPPC/DPPG Mixed Monolayers at Constant Area

After the monolayer is compressed to an initial surface pressure ($\pi_{0}$ = 5 mN/m, 15 mN/m, and 25 mN/m), the total area of the monolayer remains constant, and the surface pressure will reach equilibrium pressure over time. The surface pressure–time (π−t) curves at constant area can be obtained to suggest the relaxation process of the monolayers [31]. The relaxation of the monolayer is caused by the reorganization of the monolayers. The most important parameter for analyzing the reorganization of the monolayers is the lifetime τ, which can be obtained by the data fitting of the $\pi /\pi_{0}-t$ curves (seen in Supplementary Materials Figure S1) by using the following equation [39,40]:

(5) $$\pi /\pi_{0}=C+ae^{-t/\tau }$$

where *C* could be defined as the normalized equilibrium pressure. A greater τ value suggests that the degree of disorder in the monolayers is higher, and the time of conformation transition is longer [39].

From Table 2, at 5 mN/m, the change in the τ value has no significant pattern. According to the minimum $C_{s}^{-1}$ (Figure 4C) at 5 mN/m, the DPPC/DPPG mixed monolayer and the AmB/DPPC/DPPG mixed monolayer have not entered the phase transition region, and they are in the liquid expanded phase. The orientation of the AmB molecule tends to be horizontal.

At 15 mN/m, when $x_{AmB}$ is less than 0.5, the τ value increases gradually with the increase in the AmB content, but when $x_{AmB}$ is 0.7 and 0.9, the τ values are smaller than those of the other components. This may be related to the phase transition of the mixed monolayer. When $x_{AmB}$ is 0.7 and 0.9, the mixed monolayer is undergoing the transition from the liquid expanded phase to the liquid condensed phase, in which the intermolecular cohesion is stronger, according to the $G^{E}$ value.

At 25 mN/m, the mixed monolayers are in the liquid condensed phase, and the distance between the molecules is small. The AmB molecular orientation is more vertical, and the tail chain of the lipid is extended. The τ value increases gradually with the increase in the molar ratio of AmB. According to the $G^{E}$ value, when $x_{AmB}$ is less than 0.5, the intermolecular interaction is attractive, and the intensity of the force decreases gradually with the increase in AmB. When $x_{AmB}$ is 0.7 and 0.9, the intermolecular force is repulsive. If the intermolecular cohesion is strong, the time of recombination for the monolayer is short; if the intermolecular cohesion is small, or mainly manifested as repulsion, the time of recombination for the monolayer is long. The analysis of the τ value is in good agreement with the analysis of the $G^{E}$ value.

### 2.5. Morphology and Height Analysis of the AmB/DPPC/DPPG Mixed Films

**Figure 5:**
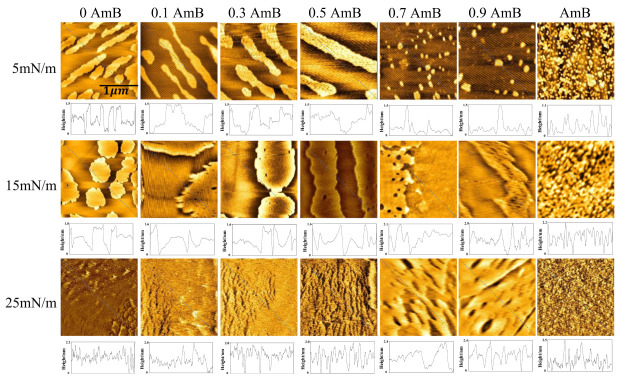
The AFM images (2 μm×2 μm) and the height curves of the DPPC/DPPG mixed monolayer with different molar radios of AmB at surface pressures of 5 mN/m, 15 mN/m, and 25 mN/m.

As shown in Figure 5, at 5 mN/m, the bright areas are shaped like long strips of island, with a height of about 1.5 nm, on the DPPC/DPPG mixed film. When $x_{AmB}$ is 0.1, 0.3, and 0.5, the shape of the bright region does not change significantly, but its area is larger than that in the absence of AmB. When $x_{AmB}$ is 0.7 and 0.9, the bright areas appear to be scattered patches. Regardless of the molar ratio of AmB, the height of the bright region on the DPPC/DPPG mixed films shows no obvious change. The pure AmB film is a relatively uniform sheet, with a height of about 1.1 nm. At 5 mN/m, the molecular axis of AmB is parallel to the interface, and the height of the AmB film is relatively low. AmB affects the morphology of the DPPC/DPPG mixed film, which depends on the molar ratio of AmB in the mixed system.

**Figure 6:**
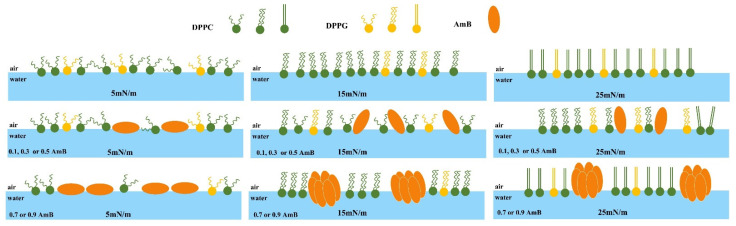
Distribution of molecules on the mixed monolayer.

At 15 mN/m, the bright region on the DPPC/DPPG mixed film is shaped like circular sheets, with a height of about 1.8 nm, similar to the results presented in a previous work [28]. This indicates that the orientation of the lipid molecules is more vertical, or the molecular chain is longer than that at 5 mN/m. At 15 mN/m, an obvious phase transition occurs for the pure AmB monolayer, according to the π−A isotherm and $C_{s}^{-1}$ analysis, and the orientation of many AmB molecules becomes more vertical. This point is verified by the AmB film’s height of about 2.1 nm. When $x_{AmB}$ is 0.1, 0.3, and 0.5, the bright regions are larger, and their height is about 1.6 nm. This may be due to the fact that the molar ratio of the AmB molecules to the lipid is no higher than 1:1, as well as the interaction of AmB with the ${PO}_{2}^{-}$ group of DPPC [40] in the monomers or dimers (Figure 6), which restricts the elongation of the molecular chains. However, when $x_{AmB}$ is 0.7 and 0.9, their height is about 2.1 nm and 2.0 nm, respectively. Due to the large molar ratio of the AmB molecules, AmB may appear in the form of a complex in the mixed system (Figure 6), and the orientation of the AmB molecules is relatively fixed, and this is not affected by its interaction with the lipid molecules.

At 25 mN/m, the DPPC/DPPG mixed film is uniform, and a small amount of flaky film is also observed with the height of about 2.2 nm. The height of the pure AmB film is about 2.5 nm. This suggests that both the lipid and AmB molecules are more inclined to horizontal or chain elongation (Figure 6). When $x_{AmB}$ is 0.1, 0.3, and 0.5, the film is shaped like a gully, and its height is about 2.0 nm. When $x_{AmB}$ is 0.7 and 0.9, the height of the mixed film is about 2.4 nm. The effect of AmB on the height of the DPPC/DPPG mixed film is similar to that at 15 mN/m.

The orientation of the AmB molecules in the DPPC/DPPG mixed monolayers varies with the surface pressures. At a high surface pressure, the AmB molecules tend to be more vertically distributed in the membrane. The orientation of AmB affects the formation of ion channels [41]. When AmB forms ion channels on the fungal membrane, it exerts its effect, but when it forms ion channels on the pulmonary surfactant membrane, it will affect the structure and function of the pulmonary surfactant membrane. During respiration, the surface pressure of the pulmonary surfactant membrane varies from 0 mN/m to 25 mN/m. When the molar ratio of AmB to lipids is greater than 1:1, AmB may aggregate into a cluster, which more easily forms ion channels, and six AmB molecules may form a single particle channel [42].

## 3. Material and Methods

### 3.1. Materials

1,2-dipalmitoyl-sn-glycero-3-phosphocholine (DPPC: purity ≥ 99%), 1,2-dipalmitoyl-sn-glycero-3-phosphoglycerol sodium (DPPG: purity ≥ 99%), and amphotericin B (AmB: purity > 80%) powder were purchased from Sigma, St. Louis, MO, USA. The AmB drug was purified before use as follows: (1) the drug powder was dissolved in high purity water, and then the solution was centrifuged for 15 min at 15,000× *g* in order to remove microcrystals of the drug still remaining in the sample; (2) the drug solution was further purified by means of HPLC using a YMC C-30 (Europe GmbH, Munich, Germany) coated phase reversed column (length 250 mm, internal diameter 4.6 mm) with 40% 2-propanol in H_2_O as a mobile phase [43]; (3) the AmB powder with a purity of 90.2% was obtained after evaporating the water. The other chemicals were of analytical grade and were used without further purification. The high purity water used in all experiments was obtained from a Milli-Q plus water purification system (18.2 MΩ/cm, Millipore, MA, USA).

The DPPC and DPPG with a molar ratio of 4:1 were fully dissolved in a chloroform/methanol (9:1, *v:v*) mixture to yield a final concentration of 0.5 mM lipid membrane-forming solution. AmB was dissolved in a 3:1 (*v:v*) mixture of dimethylformamide and 1 M HCl to yield a final concentration of 0.5 mM.

### 3.2. Langmuir Technique

An air–water interface can be provided by a Langmuir trough with the size 323 mm × 75 mm × 5 mm (KSV-Minitrough, Oulu, Finland) to study the behavior of the monolayer. Two Teflon barriers on the trough could symmetrically compress or expand the monolayer at the air–water interface at a desired rate. A Wilhelmy plate tensiometer using filter paper (10 mm × 30 mm × 0.15 mm) can be used as a pressure sensor to monitor the surface pressure, with the accuracy of 0.1 mN/m.

The specific experimental operation method was as follows. Firstly, the Teflon trough was washed with ethanol and rinsed with purified water. Then, 200 mL of 20 mM HEPES (N-2-hydroxyethylpiperazine-N-2-ethanesulfonic acid, pH 7.0) buffer was added to the trough to maintain the stability of the pH value in the DPPC/DPPG mixed monolayer environment. The 20 μL DPPC/DPPG mixed solution or 20 μL pure AmB solution was deposited at the air–water interface by a Hamilton microsyringe. After 15 min, the monolayer was compressed with a barrier speed of 7 mm/min. The surface pressure–mean molecular area isotherm of the DPPC/DPPG mixed monolayer or pure AmB monolayer was recorded during the interface compression process. To quantitatively study the influence of AmB on the interface behavior of the DPPC/DPPG mixed monolayer, the molar ratio of the AmB in the mixing system was set to 0.1, 0.3, 0.5, 0.7, and 0.9, respectively. The DPPC/DPPG mixed solution and the AmB solution were added successively onto the interface. The total volume was 20 μL. According to the above method, the surface pressure–mean molecular area isotherms of the AmB/DPPC/DPPG mixed monolayers were obtained. The monolayer was compressed to cause the surface pressure to reach 5 mN/m, 15 mN/m, and 25 mN/m, respectively, keeping the area of the monolayer constant. The surface pressure–time (π−t) curves of the monolayers were recorded.

Each experiment was repeated three times to confirm the reproducibility of the isotherm measurements. For all the experiments, the temperature was maintained at 35.0 ± 0.5 °C by an external circulator.

### 3.3. Atomic Force Microscopy

After dipping a fresh mica sheet vertically below the air–water interface, a monolayer was prepared at the interface. The monolayer was compressed to 5 mN/m, 15 mN/m, or 25 mN/m, and the surface pressure was kept constant. The fresh mica sheet was pulled upward along the vertical direction at a speed of 5 mm/s. In this way, the monolayer at the air–water interface was transferred onto the mica sheet, forming the Langmuir–Blodgett (LB) film. The structure of the LB film on the mica surface was observed by atomic force microscopy (Shimadzu, Kyoto, Japan). The AFM images were obtained in the contacting mode using a silicon nitride pyramidal tip mounted on a 100 μm long cantilever, with a force constant of 0.1 N/m.

## 4. Conclusions

In this work, the effect of AmB on the thermodynamic properties and surface morphology of the pulmonary surfactant model monolayer during respiration was studied. The parameters $A_{\infty }$, $\pi_{C}$, and $A_{C}$ show a consistent pattern, and they decrease gradually with the increase in the molar ratio of AmB. The intermolecular interaction between AmB and the pulmonary surfactant lipids is attractive in most instances, according to the $G^{E}$ value. However, when $x_{AmB}$ is greater than 0.5, the intermolecular interaction is repulsive at a surface pressure higher than 15 mN/m. The analysis of the τ value is in good agreement with the analysis of the $G^{E}$ value. The effect of AmB on the stability of the DPPC/DPPG monolayer is directly related to the molar ratio of AmB in the system. When the molar ratio of AmB is 0.7 and 0.9, the AmB/DPPC/DPPG mixed monolayer is the most stable at 15 mN/m. The height of the mixed monolayer increased when $x_{AmB}$ was 0.7 and 0.9, but decreased when $x_{AmB}$ was 0.1, 0.3, and 0.5 and 15 mN/m and 25 mN. When the molar ratio of AmB is large, it may interact with the pulmonary surfactant membranes in the form of complex. Although there are still differences between the DPPC/DPPG mixed monolayers and the real pulmonary surfactant model monolayers, the results of this work well reflect the possible interaction between AmB and the pulmonary surfactant model monolayer during respiration. This is helpful for understanding the effects of different doses of AmB drugs on the alveolar surface membrane when they penetrate the surfactant monolayer during respiration.

## Figures and Tables

**Table 1:** The $A_{L}$, $A_{\infty }$, $\pi_{C}$, and $A_{C}$ values of the AmB/DPPC/DPPG mixed monolayers at the air–water interface.

Monolayer	$A_{L}/{Å}^{2}$	$A_{\infty }/{Å}^{2}$	$\pi_{C}/mN/m$	$A_{C}/{Å}^{2}$
DPPC/DPPG	136.76 ± 0.24	61.87± 0.34	54.01± 0.30	44.29 ± 0.27
0.1 AmB	142.23 ± 0.14	61.63 ± 0.21	54.08 ± 0.20	39.76 ± 0.44
0.3 AmB	151.62 ± 0.15	56.55 ± 0.20	54.15 ± 0.20	33.93 ± 0.23
0.5 AmB	138.55 ± 0.42	50.98 ± 0.55	53.93 ± 0.52	24.85 ± 0.47
0.7 AmB	153.52 ± 0.41	40.79 ± 0.38	50.04 ± 0.29	16.9 ± 0.40
0.9 AmB	146.14 ± 0.54	36.55 ± 0.40	48.61 ± 0.46	6.85 ± 0.52
AmB	175.18 ± 0.25	32.99 ± 0.33	42.29 ± 0.32	2.69 ± 0.22

**Table 2:** The values of $C,a,\tau ,r^{2}$ obtained by fitting the decay curves to a single-exponential equation.

Monolayer	Surface Pressure of the Monolayer											
	5 mN/m				15 mN/m				25 mN/m			
	C	a	τ	$r^{2}$	C	a	τ	$r^{2}$	C	a	τ	$r^{2}$
DPPC/DPPG	0.57	0.35	890.98	0.99	0.68	0.26	820.27	0.99	0.77	0.19	811.31	0.98
0.1 AmB	0.12	0.87	1273.35	0.98	0.77	0.18	910.27	0.99	0.45	0.47	902.89	0.99
0.3 AmB	0.38	0.52	1322.71	0.99	0.53	0.38	963.31	0.99	0.11	0.87	964.05	0.99
0.5 AmB	0.64	0.29	1121.28	0.98	0.57	0.36	1057.92	0.99	0.47	0.43	1005.76	0.98
0.7 AmB	0.25	0.64	1057.31	0.99	0.69	0.23	599.06	0.98	0.74	0.21	1314.97	0.99
0.9 AmB	0.52	0.41	1538.51	0.98	0.80	0.10	301.74	0.98	0.62	0.32	1388.21	0.99

## Data Availability

Not applicable.

## Supplementary Materials

The following are available online at https://www.mdpi.com/article/10.3390/molecules28124840/s1, Figure S1: The $\pi /\pi_{0}-t$ curves of the AmB/DPPC/DPPG mixed monolayer at 5 mN/m, 15 mN/m, and 25 mN/m.

## Sample Availability

Samples of the compounds InP/ZnS QDs are available from the authors.
