# Supplementary figures and images for: Effect of Amphotericin B on the Thermodynamic Properties and Surface Morphology of the Pulmonary Surfactant Model Monolayer during Respiration
Source: Molecules. 2023 Jun 18;28(12):4840. doi: 10.3390/molecules28124840 (PMC10305212; doi:10.3390/molecules28124840)

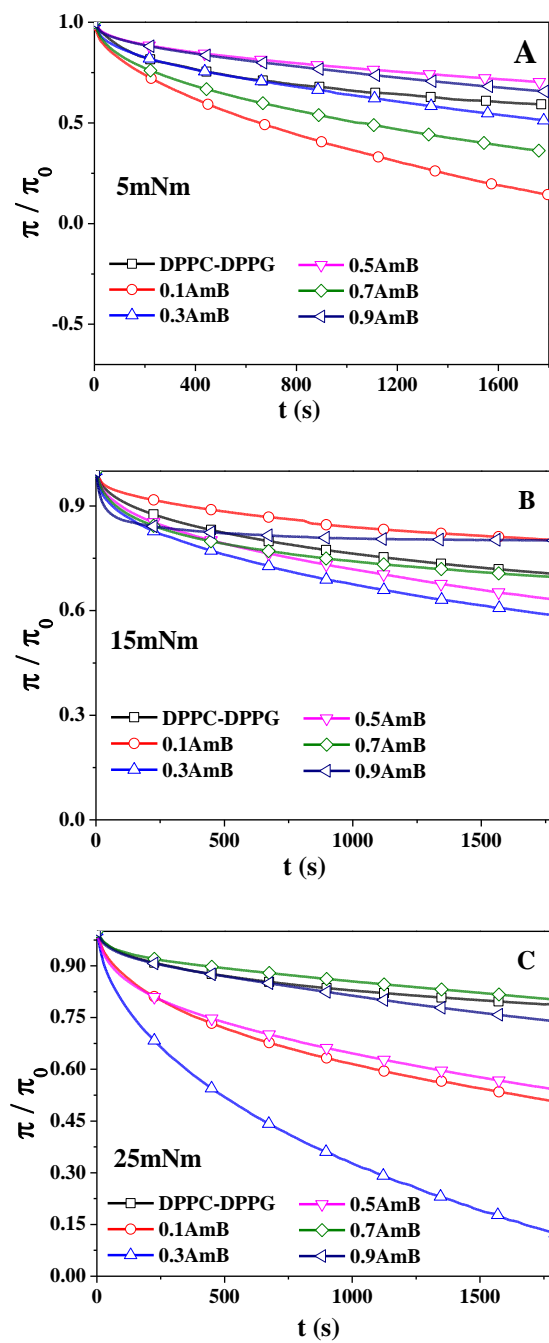

Figure S1 The  $\pi / \pi_0 - t$  curves of the AmB/DPPC/DPPG mixed monolayer at 5mN/m, 15mN/m and 25mN/m.

Supplement: Supplementary file 1 [file molecules-28-04840-s001.zip › molecules-2441331-supplementary.pdf]
